# Strategies for Designing and Monitoring Malaria Vaccines Targeting Diverse Antigens

**DOI:** 10.3389/fimmu.2014.00359

**Published:** 2014-07-28

**Authors:** Alyssa E. Barry, Alicia Arnott

**Affiliations:** ^1^Division of Infection and Immunity, Walter and Eliza Hall Institute of Medical Research, Parkville, VIC, Australia; ^2^Department of Medical Biology, The University of Melbourne, Parkville, VIC, Australia

**Keywords:** *Plasmodium falciparum*, *Plasmodium vivax*, malaria, vaccine, strain, diversity, polymorphism, clinical trials

## Abstract

After more than 50 years of intensive research and development, only one malaria vaccine candidate, “RTS,S,” has progressed to Phase 3 clinical trials. Despite only partial efficacy, this candidate is now forecast to become the first licensed malaria vaccine. Hence, more efficacious second-generation malaria vaccines that can significantly reduce transmission are urgently needed. This review will focus on a major obstacle hindering development of effective malaria vaccines: parasite antigenic diversity. Despite extensive genetic diversity in leading candidate antigens, vaccines have been and continue to be formulated using recombinant antigens representing only one or two strains. These vaccine strains represent only a small fraction of the diversity circulating in natural parasite populations, leading to escape of non-vaccine strains and challenging investigators’ abilities to measure strain-specific efficacy in vaccine trials. Novel strategies are needed to overcome antigenic diversity in order for vaccine development to succeed. Many studies have now cataloged the global diversity of leading *Plasmodium falciparum* and *Plasmodium vivax* vaccine antigens. In this review, we describe how population genetic approaches can be applied to this rich data source to predict the alleles that best represent antigenic diversity, polymorphisms that contribute to it, and to identify key polymorphisms associated with antigenic escape. We also suggest an approach to summarize the known global diversity of a given antigen to predict antigenic diversity, how to select variants that best represent the strains circulating in natural parasite populations and how to investigate the strain-specific efficacy of vaccine trials. Use of these strategies in the design and monitoring of vaccine trials will not only shed light on the contribution of genetic diversity to the antigenic diversity of malaria, but will also maximize the potential of future malaria vaccine candidates.

## Introduction

Malaria is the most devastating parasitic disease afflicting humankind. The disease results from infection with protozoan parasites of the genus, *Plasmodium* and is transmitted by female anophelene mosquitoes. Of the 3.4 billion people in 108 countries at risk of malaria, 1.2 billion are at high risk of disease. In 2012, it was estimated that this disease caused 2000 deaths per day, the majority (77%) being children <5 years of age in sub-Saharan Africa infected with *Plasmodium falciparum*, the most virulent of the five known human malaria parasites (1, 2). In addition to this enormous heath toll, malaria exerts a heavy economic burden contributing to the cycle of poverty in many resource-limited settings (3). Although less lethal than *P. falciparum*, the majority of malaria infections occurring outside of sub-Saharan Africa are caused by *Plasmodium vivax*, with as many as 2.3 billion people at risk of infection (4). Several unique features of *P. vivax* biology, including its dormant stage in the human liver, make it more resistant to malaria elimination. As a result, *P. vivax* is predicted to present the ultimate obstacle to malaria elimination in endemic countries (5). Nevertheless, research into this parasite lags far behind that of *P. falciparum* due to its relatively recent recognition as a serious threat to global public health and lack of a viable long term *in vitro* culture system (4, 6).

Intensified malaria control efforts, supported by the Roll Back Malaria campaign, have resulted in a 42% decrease in malaria deaths worldwide in the last decade and many previously endemic countries have now shifted from controlling malaria to an elimination agenda (1). In 2007, encouraged by the stunning impact of this campaign, major funding bodies united to issue the ultimate challenge, to eradicate malaria globally by progressive malaria elimination from different countries and regions (3, 7). From past malaria eradication attempts, it is clear that in order for this ambitious goal to be achieved, malaria transmission must be permanently interrupted. Interventions that reduce the parasite reservoir, limit the rate at which infections are spread and the duration of time that a human or mosquito host is infectious are therefore urgently needed (8). In concert with other malaria control interventions, this could be achieved with the development of a broadly effective malaria vaccine.

Malaria parasites are ancient organisms with abundant genetic polymorphisms, much of which have evolved to escape host immune responses and thus presents a major obstacle to the development of a vaccine that provides broad protection against all, or at least the majority of strains (9). As with other pathogens, the challenge in developing an effective malaria vaccine will be to differentiate between diversity that is associated with immune escape and cross protection, and that which has no bearing on the immune response, having simply accumulated over time through genetic drift or through adaptation to diverse host environments (9). To date, the polymorphisms in malaria antigens targeted by functionally important antibodies remain poorly characterized (10). Very little is known of how sequence polymorphisms relate to antigenic diversity or the potential for polymorphisms to mediate vaccine escape for *Plasmodium* spp. (11). The key to success with other pathogens has been the identification of immunologically relevant diversity. This has been achieved by performing population genetic and structural studies to identify functionally relevant polymorphisms, followed by molecular epidemiological surveys or *in vitro* functional studies prior to development and testing of vaccines (9). Narrowing the focus to immunologically relevant polymorphisms would greatly reduce the diversity that must be considered when developing multivalent malaria vaccines covering a broad range of strains (2, 9, 12) (Box 1).

Box 1 **Glossary of terms.**

***Strain:*** a parasite variant that is genetically unique and induces specific immune responses against one or more of its antigens.

***Isolate:*** a parasite specimen derived from an infected individual that has been either adapted for *in vitro* culture or used directly for experiments. Different isolates from the same population may contain parasites that are genetically identical at one or more loci. Individual isolates may also contain one or more genetically distinct parasites if they have been collected from an individual with multiple infections.

***Clone:*** a genetically homogeneous parasite isolate.

***Polymorphism:*** variation in the population at a particular nucleotide or amino acid residue.

***Allele:*** one variant of a particular genetic locus. It can refer to individual polymorphic sites within a nucleotide or amino acid sequence, or the combination of all polymorphic sites in a gene or gene region (known as the haplotype).

***Haplotype:*** a combination of alleles in a gene or gene region carried by a particular parasite clone.

***Serotype:*** a haplotype from a gene or gene region that is antigenically unique and induces strain-specific responses.

***Monovalent vaccine:*** a vaccine containing only one distinct antigen or one allele of the same antigen.

***Multivalent vaccine:*** a vaccine containing two or more distinct antigens, or two or more alleles of the same antigen.

## Malaria Vaccines: Past, Present, and Future

A long lasting, broadly efficacious malaria vaccine would be the most sustainable approach to control and eventually eradicate malaria. That a malaria vaccine may be feasible is strongly supported by the fact that people living in malaria endemic areas develop protective immunity against malaria symptoms during childhood (13). By adulthood, decreases in the prevalence of infection and density of parasitemia are achieved indicating that this immunity eventually provides some protection against infection (14, 15). Passive transfer of immunoglobulin from hyper-immune African adults to non-immune children with severe malaria was shown to have curative properties, demonstrating that antibody responses are largely responsible for protection against clinical disease (16). Furthermore, vaccination of rodent and primate models with recombinant parasite antigens elicits high antibody titers that are associated with protection against subsequent malaria challenge (17–20). Although the development of malaria vaccines has been an active focus of the malaria research community over the last 50 years, a vaccine remains a missing component of malaria control and elimination strategies (21).

Despite numerous promising malaria vaccine candidates and several partially successful malaria vaccine trials (22–25), short-lived protection, limited funding, and a lack of key technologies has hampered further testing and the scale-up of clinical trials of novel malaria vaccine candidates. The first successful malaria vaccine trial, based on a “whole parasite” approach, was conducted in humans in the 1950s. Vaccination with irradiated sporozoites was shown to protect against both homologous and heterologous challenge in humans (26, 27). However, the need for large-scale production prevented further development of this approach. With the advent of molecular technologies in the 1980s, the focus shifted to so-called “subunit” approaches, including highly immunogenic parasite antigens as targets such as the circumsporozoite protein (CSP), which was identified as the parasite antigenic determinant targeted by immune responses induced by the sporozoite vaccine (28). Following these early studies, and on the back of highly promising pre-clinical studies, many small-scale subunit vaccine trials were conducted in humans. Efficacy was highly variable with many candidates demonstrating no protective effect, however, there were some promising candidates identified that continue to be further developed today (21), and these are discussed in more detail below.

In the last decade, there has been an attempt to assist and accelerate development of a malaria vaccine with the establishment of the PATH Malaria Vaccine Initiative (MVI), which has greatly progressed the evaluation and identification of promising malaria vaccine candidates. To further focus and unite the global vaccine effort, in 2006 the World Health Organization (WHO) launched the first malaria vaccine technology roadmap with the landmark goal:

By 2015, develop and license a first generation malaria vaccine that has a protective efficacy of more than 50% against severe disease and death and lasts more than one year (29).

Currently, a single vaccine candidate is on track to meet this goal. Known as “RTS,S,” this vaccine is based on the repeat region and T-cell epitopes of CSP. RTS,S is the subject of a large multicentre Phase 3 trial involving more than 15,000 children over 11 sites in sub-Saharan Africa. The full results of the trial have not yet been published; however, after three doses, clinical malaria cases during the first 18 months of follow-up decreased by an estimated 46% (severe disease by 36%) in children 5–17 months of age at first vaccination, and 27% (severe disease by 15%) in infants aged 6–12 weeks of age at first vaccination (30), therefore, RTS,S efficacy is approaching the above-mentioned WHO criteria for a first generation malaria vaccine. As a result, this vaccine is likely to be licensed in 2015 for use in young African children and could lead to significant decreases in malaria morbidity and mortality in this high-risk population. Despite this positive outlook, it is cautioned that this vaccine is only partially protective against disease and wanes over time. New, second-generation vaccines will need to have major improvements in efficacy to meet the challenges ahead (10).

Recognizing the changing epidemiology of malaria in the context of a shrinking global malaria map and a shift in populations most at risk of infection (5), as well as the need for vaccines with higher efficacies than RTS,S if malaria elimination is to be achieved, the goals of the Malaria Vaccine Technology Roadmap were recently reset with two major objectives:

By 2030, license vaccines targeting *P. falciparum* and *P. vivax* that encompass: (i) development of second-generation malaria vaccines that provide a protective efficacy of more than 75% against clinical (mild and severe) malaria … and (ii) development of malaria vaccines that reduce transmission of the parasite and thereby reduce the incidence of human infection … (31).

To achieve these new goals, a better understanding of the minimal requirements and mechanisms underlying development of immunity against *P. falciparum* and *P. vivax* (10, 12) as well as knowledge regarding the factors that influence transmission of both species, will be essential. As the development of vaccines against *P. vivax* is also a major goal, it will be important to consider the distinct features of this species, which underscores the need to intensify research efforts into this relatively neglected parasite.

## Approaches to Malaria Vaccine Development

Malaria parasites are complex eukaryotes comprised of many antigenic targets. It has been suggested that vaccines may need to be as complex as the parasite itself (32) and therefore there has been considerable interest in the whole parasite approach. As mentioned above, irradiated live sporozoite vaccinations have shown great success in clinical trials (27, 33, 34). Currently, different methods are being used to attenuate sporozoite stages including chemical and genetic modification, and these have been reviewed elsewhere (35). However, there are some challenges to overcome in addition to the technical difficulties and high costs associated with scaling up production including the dose required to induce long-lasting protective immunity, transport, and storage in the absence of a reliable cold chain for distribution to at risk populations (36). There is also a risk of reversion to virulence (12). Hence the alternative subunit vaccine approach continues to be vigorously evaluated (37). As indicated above, this involves individual recombinant parasite proteins administered as monovalent preparations or combinations of multiple proteins together with different vectors and adjuvants that enhance the immune response. As the majority of clinical trials conducted to date have been based on subunit vaccines, this rest of this review will focus on the development of this class of malaria vaccines and the challenges associated with this approach.

**Figure 1:**
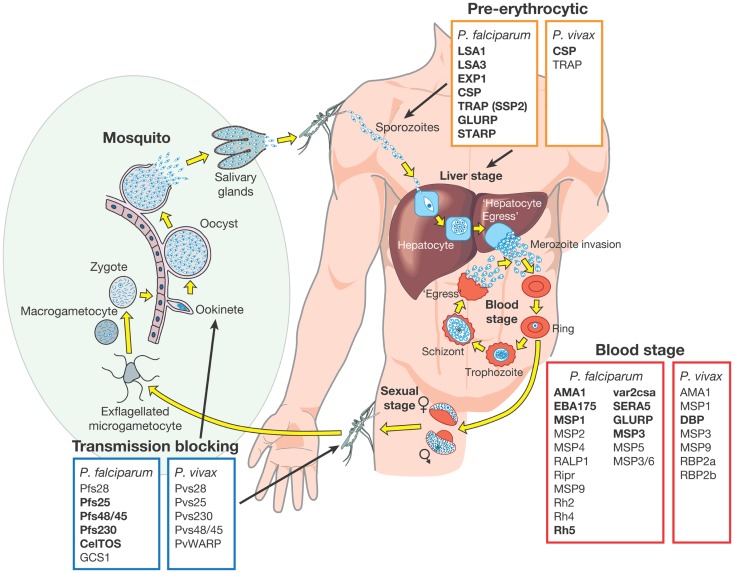
**Malaria vaccine candidate antigens are shown**. All candidate antigens for *Plasmodium falciparum* and *Plasmodium vivax* are superimposed on the *Plasmodium* lifecycle, to indicate the category of malaria vaccine being developed and the lifecycle stage targeted. Antigens indicated in bold are those that are currently being evaluated in pre-clinical trials or have entered at least Phase 1 clinical trials according to the WHO malaria vaccine rainbow tables (50). The *P. vivax* latent stages known as “hyponozoites” are not shown but these occur in the liver stage.

Several highly abundant parasite proteins were identified as targets of natural immunity many years ago but in recent years the list of possible candidates has expanded. These candidates have been extensively validated in pre-clinical studies using different approaches including the measurement of inhibitory antibody responses in *in vitro* growth and invasion assays (short term culture only for *P. vivax*) (18, 38–41) and by vaccinating animal models followed by challenge infections (17, 18, 42–45). Subunit vaccines have been or are being developed based on these advanced candidate antigens, which are expressed in almost every stage of the parasite lifecycle. They have been classified into one of three different groups based on the target lifecycle stage (Figure 1) (46):

(i) *pre-erythrocytic vaccines*: these vaccines aim to prevent infection by targeting the infective stage, known as the sporozoite (e.g., RTS,S). Alternatively, pre-erythrocytic vaccines can target antigens expressed by liver stage parasites to prevent the emergence of merozoites into the bloodstream, the stage of infection responsible for the clinical symptoms of malaria infection (Figure 1). The risk associated with targeting sporozoite antigens is that the antigen dose inoculated during a natural infection is very low, with only a small number of sporozoites injected by the vector (~20), and this may not be sufficient to elicit an effective host immune response. Additionally, only one sporozoite needs to escape the vaccine-mediated immune response and invade liver cells for ~10,000 infectious merozoites to be produced, resulting in blood stage infection and clinical disease (47).
(ii) *blood stage vaccines*: the vast majority of malaria vaccine candidates are designed to protect against the blood stage of infection (Figure 1). Since all of the symptoms of malaria occur during this stage, the majority of vaccines targeting antigens expressed during the blood stage are designed primarily to prevent disease. One approach is to target merozoite antigens to prevent red blood cell invasion and reduce the density and prevalence of parasites in the infected host (Figure 1). In principle, this reduction in parasite density may also reduce the density of transmission forms, known as gametocytes (i.e., the sexual stage of the parasite transmitted from human to mosquito host) (Figure 1). In addition to preventing clinical disease, an effective blood stage vaccine that reduces parasite density may therefore also contribute to reducing malaria transmission (10, 46, 48). Approaches are also being developed to target the major surface protein expressed on the *P. falciparum* infected red blood cell known as erythrocyte membrane protein 1 (PfEMP1). PfEMP1 mediates adhesion to host cells, a mechanism that is associated with severe malaria [reviewed by Hviid (49)].
(iii) *transmission-blocking vaccines*: the aim of transmission-blocking vaccines is to target antigens expressed during lifecycle stages in the mosquito host (e.g., gametocyte or oocyst antigens) (Figure 1). Although these vaccines would not directly prevent infection or clinical disease, they would greatly assist elimination efforts to prevent the onward transmission of infections that may be imported into an elimination zone (10, 46).

Development of a vaccine against *P. falciparum* is well advanced with 31 promising antigens identified (Figure 1). Currently, 27 subunit candidates comprising different domains and alleles for 22 different antigens are being tested in pre-clinical or clinical trials (50) (Figure 1; Table 1). However, the majority of candidates tested in clinical trials so far have been based on different formulations and regions of a handful of antigens identified many years ago, including CSP (51), the liver stage antigen 1 (LSA1) (52), thrombospondin-related antigen (TRAP, also known as sporozoite surface protein 2, SSP2) (53–55), merozoite surface protein 1 (MSP1) (56), MSP2 (57), MSP3 (58), and apical membrane antigen 1 (AMA1) (42, 59). As some of these antigens have shown promising pre-clinical profiles yet limited efficacy in human trials, they have been tested in many different formulations over the years (21, 60). For example, the most advanced blood stage antigens, AMA1 and MSP1, naturally induce protective immune responses (61–63), demonstrated using *in vitro* inhibition assays (64) and by vaccination of animal models (17, 18, 20, 65). However in humans, only limited clinical efficacy has been observed (66, 67). Similarly, CSP, the major component of the RTS,S vaccine, is the major surface antigen on the sporozoite surface, yet provides only partial and short-lived protection against the blood stage symptoms of malaria following vaccination of human volunteers. It does not protect against infection *per se* as would be expected by a pre-erythrocytic vaccine and therefore the precise mechanism of protection is not well understood (23, 68). These “historical” vaccine candidates are much further down the development pipeline (21) than more recently identified candidates such as the invasion ligands, the 175 kDa erythrocyte binding antigen (EBA175) (69), reticulocyte binding homolog 5 (RH5) (70, 71), and *P. vivax* Duffy binding protein (DBP) (72). In recent years, the *var2csa* variant of PfEMP1, which is the major parasite ligand involved in placental adhesion during pregnancy malaria (73); and the gametocyte antigens, Pfs25 and Pfs48/45 (74) representing promising transmission-blocking targets, have also come to the forefront. The elucidation of the complete parasite genome (75) has further advanced vaccine development by enabling identification of scores of novel antigens. Subsequent antigenic credentialing through functional studies (76), proteomic and immunological screening (63, 77–80), and population genetic analyses (81, 82), has resulted in prioritization of several of these promising candidates for further development.

**Table 1:** **Diversity of malaria vaccine candidate antigens currently in clinical trials based on the WHO rainbow tables (50)**.

Antigen	Lifecycle stage	Domain analyzed	Number of [-0.5pt] continents [-0.5pt] surveyed	Number of [-0.5pt] countries [-0.5pt] surveyed	Number of isolates [-0.5pt] sampled (range)	Number of haplotypes [-0.5pt] identified (range)	Reference
**PLASMODIUM FALCIPARUM**							
CSP	Sporozoite	C-terminal	3	13	604 (9–143)	71 (3–20)	(2)
			1	1	157	n.r. (13–34)	(83)
			1	1	100	57	(84)
			3	17	1339 (9–336)	117 (1–40)	(85)
		Full length	2	7	485	n.r.	(86)
STARP	Sporozoite	Full length	1	1*	134 (10–24)	24	(87)
TRAP	Sporozoite	N-terminal	2	3	100 (8–48)	84 (8–37)	(2)
LSA1	Liver stage	N-terminal	3	4	74 (10–22)	13 (3–7)	(2)
GLURP	Sporozoite/gametocyte	Region 0	3	3	48 (9–11)	22 (2–9)	(2)
		Region 0 and 2	1	1	77 (R0); 79 (R2)	n.r.	(88)
AMA1	Merozoite	Domain I	3	11	572 (8–162)	181 (6–68)	(2)
			1	1	193 (9–100)	139 (6–58)	(89)
		Full length	2	7	459	n.r.	(86)
			1	1	21	11	(90)
			1	1	129	78	(91)
			1	1	315	168	(92)
EBA175	Merozoite	Region II	2	3	135 (30–48)	51 (15–23)	(2)
MSP1	Merozoite	MSP1_19_	3	11	2237 (18–1368)	20 (1–15)	(2)
			1	1	136	12	(83)
			1	1	61 (9–15)	5	(93)
			1	1	300	19	(94)
		Block 2	1	1	35	23	(95)
			1	1	36	13	(96)
			1	1	128	14	(97)
		Full length	2	7	404	n.r.	(86)
MSP2	Merozoite	Blocks 2 and 3	2	3	392 (n.d)	275 (n.r.)	(2)
		Block 3	1	1	148	22	(97)
MSP3	Merozoite	Repeat region	2	2	124 (75–86)	21 (9–12)	(2)
MSP4	Merozoite	Full length	2	4	142 (12–42)	47 (9–23)	(2)
MSP3/6	Merozoite		1	2	117 (51–66)	n.r.	(81, 82)
Rh2	Merozoite	Binding region	1	1	33 (15)	n.r. (13)	(98)
Rh4	Merozoite	Binding region	1	1	23 (12)	9 (4)	(99)
RH5	Merozoite	Full length	3	6	227 (21–125)	n.r.	(100)
Pfs48/45	Gametocyte	Full length	3	4	55 (9–15)	19 (2–8)	(2)
Pfs28	Ookinete	No population data available					
Pfs25	Ookinete	Full length	2	2	41	n.r.	(101)
*var2csa*	Trophozoite	DBL3	2	3	124 (15–54)	n.r.	(102)
		DBL3X	1	1	108	79	(103)
		DBL5	1	2	70	n.r.	(104)
*var*	Trophozoite	DBLalpha	3	4	29–42 (32)	140–666 (449)**	(105)
SERA5	Trophozoite/schizont	Exon II–IV	4	9	445 (39–80)	133 (3–44)	(106)
**PLASMODIUM VIVAX**							
CSP	Pre-eryth	Central repeat (CR)	2	2	168 (31–137)	n.r. (13–25)	(107, 108)
			3	9	194	76 (5–23)	(109)
			1	1	84	23	(95)
DBP	Merozoite	Region II	2	8	675 (11–123)	n.r. (9–73)	(110)
			3	9	707 (11–200)	150 (8–59)	(111)
			1	1	63	16	(112)
			1	1	70	13	(113)
			1	1	22	8	(114)
			1	1	54	12	(115)
			3	7	402 (9–122)	138 (7–56)	(116)

*Also included a small number of strains from Brazil, Indonesia, Tanzania and Kenya.

**Predicted total number of *var* alleles is much higher: 232–7565 (3564).

Research on potential *P. vivax* vaccine candidates lags far behind that of *P. falciparum*. Currently, only two *P. vivax* vaccine candidates (based on PvCSP and PvDBP) have shown promise in pre-clinical studies and only one candidate has reached Phase 1a clinical trials (50) (Figure 1). If the new goals of the Malaria Vaccine Technology Roadmap are to be realized, significantly more resources need to be invested in identifying and validating promising *P. vivax* vaccine candidates for progression to clinical trials.

## Parasite Diversity: A Major Obstacle to Malaria Vaccine Development

One often quoted explanation for the variable efficacy of subunit malaria vaccine candidates is parasite genetic diversity (2, 9, 12, 37) and the strain-specific nature of immunity to diverse parasite antigens (15, 117, 118). Theoretically, a malaria “strain” can be defined as a parasite variant that is genetically distinct and induces specific immune responses against one or more antigens (Box 1). However, exactly what defines a *Plasmodium* strain is not fully understood (119) because this definition becomes exceedingly complex when the whole parasite is considered. In the context of subunit vaccines, the term “strain” refers to the parasite isolate from which the vaccine antigen is derived, while the actual genetic variant of that antigen is known as the “allele” or “haplotype” (Box 1). The inclusion of only one allele in a subunit vaccine formulation elicits responses only against similar, cross-reactive alleles (the “serotype,” Box 1) and runs the risk of selection for non-vaccine strains in the vaccinated host population, as discussed in detail below (22). Indeed, natural parasite populations have large numbers of alleles or haplotypes for single copy antigens, such as AMA1 and MSP1 (2, 11) (Table 1). However for PfEMP1, which is encoded by as many as 60 different genes per parasite genome, there are hundreds to thousands of distinct alleles even within local geographic areas <10 km^2^ (105). Extensive parasite antigenic diversity explains the slow development of naturally acquired immunity (120) with repeated exposure over several years necessary to build up a large repertoire of antibodies to the different serotypes circulating in an endemic area (14, 121). Given the high diversity of the available vaccine candidates (Table 1), a broadly effective malaria vaccine may need to be multivalent, comprising multiple alleles (or haplotypes) for a given polymorphic antigen (12), much like the vaccine approaches used to successfully combat other highly polymorphic pathogens such as influenza A and human papillomavirus.

On the other hand, some *Plasmodium* antigens are relatively conserved, such as RH5 (100), or have highly conserved functional regions that vaccine-developers may be able to exploit such as the AMA1 receptor-binding pocket (122, 123). Furthermore, antibodies against major surface antigens cross-react with different parasite strains including those from different geographic areas suggesting that conserved epitopes exist (65, 124). It has therefore been proposed that using a panel of peptides containing conserved epitopes would be one approach to induce immune responses that avoid dominant polymorphic epitopes (125).

An important priority in malaria vaccine development is therefore to not only confirm the diversity circulating in the target parasite population but also to understand the contribution of *genetic* (allelic/haplotypic) diversity to the *antigenic* (serotype) diversity that is relevant to malaria vaccine design for each candidate antigen (48). Whilst there are indeed multiple diverse alleles of many candidate antigens circulating within distinct populations, not all polymorphisms, will mediate antigenic escape, hence these must be identified and targeted for vaccine design. However, the relationship between allele and serotype has been dissected for only one candidate, AMA1 (126). More rigorous investigation of available candidates as well as the identification of novel relatively conserved antigenic targets is therefore absolutely required to develop a framework for selection and to prioritize antigens for further development as vaccine candidates.

## Diversity-Covering Vaccine Approaches

Although well established, the extreme diversity of leading candidate antigens has rarely been considered when developing and testing candidate malaria vaccines [reviewed by Barry et al. (2)]. The majority of subunit vaccine candidates tested in clinical trials have been monovalent. Moreover, all vaccine candidates have been based on alleles from a handful of parasite isolates such as *3D7, FC27, FUP*, and *FVO* for *P. falciparum*, and *Sal1* for *P. vivax*, that have been propagated for decades *in vitro* (or in primate models for *P. vivax*), and poorly reflect the parasite strains circulating in natural populations (2, 110, 127–129). As a result, many malaria vaccine candidates do not adequately cover the diversity observed in natural parasite populations and this could explain the poor clinical efficacy observed in vaccine trials where efficacy endpoints include infection with any strain (21, 66, 127). A multivalent malaria vaccine comprised of multiple serotypes may perform better as it would be designed to protect against a wide range of parasite strains. However, for almost all malaria vaccine candidates, the polymorphisms that define the serotypes and the number of alleles that should be incorporated into a malaria vaccine to cover serotype diversity remain unknown.

Supporting the argument for a multivalent vaccine strategy, vaccine candidates based on a single allele for specific antigens have demonstrated more strongly protective responses when strain-specific endpoints (i.e., infection with a strain carrying the vaccine allele) have been measured as compared to standard endpoints (i.e., infection with any strain). One of the most successful vaccine trials conducted to date was that of the “Combination B” vaccine, conducted in 120 Papua New Guinean children. This vaccine contained only the *3D7* allele of MSP1, MSP2, and the ring-associated erythrocyte antigen (RESA), however, it resulted in a 62% reduction in parasite density in vaccinated children compared to those that received the placebo (22). MSP2 contains a central complex tandem repeat region and many different alleles that vary in size, however all alleles fall into two major families that form different serotypes (*3D7* and *FC27*) (130). Interestingly, at the time of vaccination, the prevalence of the *3D7*-type alleles was between 23 and 50% within each of the treatment groups, which could explain the high overall efficacy. Furthermore, vaccinees were less frequently infected and had a lower rate of clinical episodes associated with *3D7*-type parasites compared to the control group (22). Similarly, volunteers from Mali vaccinated with FMP2.1, which is based on the *3D7* allele of AMA1, had a much higher risk of non-*3D7* infections (64%) (131) than any infection (20%) based on residues in the AMA1 cluster 1 loop (c1L) (66). The results of these trials highlight the danger of vaccine-induced selection pressure and its consequences for morbidity, and strongly argue for developing vaccines covering major serotypes circulating in natural parasite populations (9, 22, 127). The frequency of vaccine or vaccine-serotypes in the target parasite population is also likely to be important for significant vaccine efficacy. The bivalent candidate, AMA1-C1 (containing *3D7* and *FVO* haplotypes) has demonstrated a lack of protective efficacy against either of the two vaccine alleles in a Phase 2b trial (92). However, this lack of observed efficacy could be explained by a low frequency of these alleles in the target parasite population and the small sample size, with only 44 sequences analyzed for both the vaccine and control groups combined. Even when the analysis of polymorphisms was narrowed to the c1L cluster of polymorphisms, which have been implicated in antigenic escape as the basis of AMA1 serotypes (126), baseline vaccine-allele frequencies were <10% indicating that much larger sample sizes would be required to observe any shift in frequency after vaccination. No other multivalent vaccine trial results are currently available, but several trials are ongoing and the malaria vaccine community awaits the final results with interest.

Multivalent combination vaccines tested in animal models have shown promising and surprising results. Vaccination with combinations of four highly diverse AMA1 alleles was shown to overcome diversity by producing a broader inhibitory response compared to single allele vaccination, thought to have occurred partly by redirecting responses to conserved epitopes (65, 132). This phenomenon, which is analogous to “original antigenic sin,” occurs because abundant, strain-specific AMA1 epitopes vary and are potentially replaced with each new infection, whereas the conserved regions remain constant. Hence, high levels of exposure to conserved epitopes with vaccination or repeated exposure during natural infections may enhance the antibody response against these regions.

Another approach to covering antigenic diversity has been to assemble all available global sequence data for an antigen target and to design a small number of synthetic protein constructs that together cover most of the diversity observed. Phase 1a and 1b trials have begun after promising pre-clinical results for a multivalent vaccine candidate (DiCo), consisting of fusion protein chimeras comprising three synthetic AMA1 molecules covering 97% of the amino acid variability, and these have been shown to elicit stronger antibody responses as a combination than alone (133). This approach has been further evaluated in pre-clinical studies together with a construct containing two allelic variants of the C-terminal 19-kDa region of merozoite surface protein 1 (MSP1_19_) fused to the DiCo construct, and again enhanced antibody responses were induced (134). Clinical trial results are not yet available for the DiCo and MSP1_19_-DiCo combination vaccines but it will be interesting to see whether the diversity-covering approach is more efficacious than the single allele approach.

## Predicting Serotypes through Population Genetic Analyses

Population genetic studies are needed to guide vaccine design, by defining the diversity of candidate antigens, to predict polymorphisms that contribute to antigenic diversity (122, 127, 135) and to investigate the geospatial distribution of predicted serotypes (136, 137). Moreover, as a continuous *in vitro* culture system is yet to be developed for *P. vivax*, epidemiological studies currently represent an important tool with which to investigate the significance of polymorphism within vaccine candidate antigens (125). This approach has been used to identify correlations between specific polymorphic sites in two leading *P. falciparum* vaccine candidates, MSP1 and AMA1, with clinical infection (127, 138).

The extensive genetic diversity of malaria vaccine candidate antigens has been demonstrated by many studies investigating genetic polymorphism in samples ranging from small numbers of geographically disparate culture-adapted isolates to large numbers of natural parasite isolates from the same local geographic area or country. These results demonstrate the high numbers of haplotypes found in natural parasite populations for many antigens (Table 1). However, there are fewer haplotypes and vaccine alleles are far more common when individual amino acid polymorphisms or limited “haplotypes” (Box 1) comprising different combinations of amino acid alleles that might form critical epitopes are considered. For example, AMA1, which has 214 amino acid haplotypes in 1 African population, has only 25 serotypes based on the c1L cluster (127), demonstrating that if the haplotype can be refined to represent only antigenic escape polymorphisms, the number of alleles required in a potential multivalent vaccine could be reduced substantially. More recent studies have suggested that the majority of the antigenic escape diversity in AMA1 may even be explained by polymorphism in just one residue (130). Importantly, in vaccine trials, a lack of knowledge of the polymorphisms that mediate antigenic escape would result in an underestimate of strain-specific vaccine efficacy. Population genetic studies are therefore critical to gain more insight into antigenic diversity and to achieve the goal of a broadly efficacious malaria vaccine.

**Figure 2:**
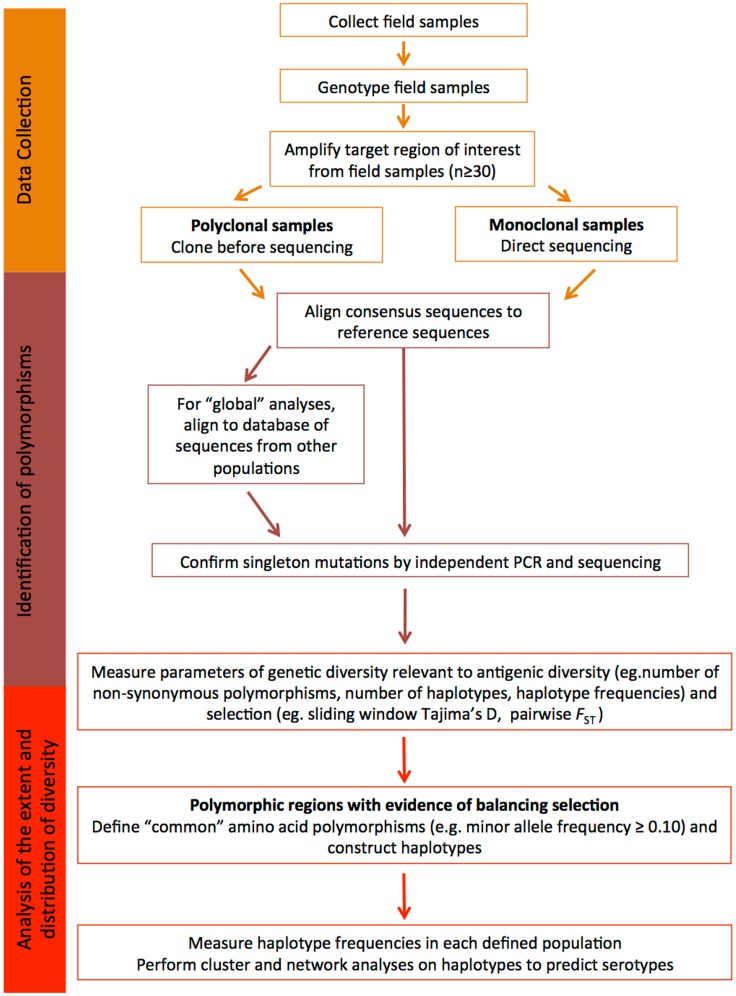
**Understanding diversity and predicting serotypes using population genetic analyses**. A flow chart describing the step-by-step methodology to define the extent and distribution of parasite diversity and to predict antigenic escape polymorphisms and serotypes (see text for more details).

Another important point to remember is that the global *P. falciparum* and *P. vivax* populations are structured into geographically distinct subpopulations, therefore, local population-level analyses are required to fully understand diversity that would be relevant to the efficacy of a malaria vaccine in a defined endemic area (139, 140). Given the large number of parasite populations that are likely to exist worldwide it may not be feasible to design vaccines for every target population and therefore a universal approach to cover diversity is needed. Below, we provide a step-by-step guide of how to identify and characterize diversity within candidate antigens that is relevant to malaria vaccine design, and this is further summarized in Figure 2.

### Data collection

In order to understand the antigenic diversity impacting on vaccine efficacy and to identify potential serotypes, the target gene or gene region encoding the candidate antigen must be amplified, sequenced, and population genetic analyses completed including the determination of regions under balancing (immune) selection. To accurately estimate natural allele frequencies, it is critical to collect sequence data from samples representing the natural parasite population of a defined geographic area. Use of samples collected in the same geographic area ensures that the diversity of the target sequence is accurately estimated for that region. As the most informative analyses of balancing selection are based on allele frequencies, it is also important that sequence data are obtained from a substantial dataset, at least 30–50 samples (81, 82). Analysis of too few sequences can result in incorrect estimates of diversity, as alleles might be under-represented in very small population samples, and this can result in skewed allele frequencies and diversity estimates.

Prior to PCR amplification, it is essential that parasite isolates are genotyped using highly polymorphic microsatellite markers to determine the number of distinct parasite clones present in the sample (141, 142). Even samples collected from areas of low malaria transmission can contain multiple clones, especially for *P. vivax* with its frequent relapses (143). If only one clone is present, sequencing can be performed directly from amplicons. If the number of field samples containing a single clone is insufficient, or polyclonal infections are common, PCR products amplified from polyclonal samples can be sub-cloned prior to sequencing. However, sub-cloning often results in artifacts (144), therefore, any novel polymorphisms should be resolved by cloning and sequencing-independent PCR products. Another approach is to perform NextGen sequencing on samples containing multiple clones, once a prohibitively expensive approach that is becoming increasingly affordable. Briefly, amplicons are sheared and adaptors added, generating a library for each sample. Libraries can be sequenced separately or as pools with the addition of sample barcodes. Reads are subsequently mapped to the relevant reference genome. The advantage of this approach is that reads are quantitative and relate to clone abundance in the sample, therefore, haplotypes of the predominant clone(s) can be computationally reconstructed (145).

### Identification of polymorphism

From the raw sequence data, consensus sequences can be obtained and compared to the reference sequence for the defined candidate. Polymorphisms identified in consensus sequences will form the basis of all downstream population genetic analyses. Quality control of sequences is therefore essential, to ensure that polymorphic sites are accurately called and that any ambiguous sites or artifacts are identified. If a clear result cannot be obtained, the sample should be removed from the analysis.

At this point, all high quality consensus sequences from a given population can be compared and basic diversity parameters determined as outlined below. In order to place the dataset in context with the known diversity, published sequences of the target region collected from other natural populations in distinct geographical areas can also be added to the analysis (e.g., from GenBank). Multiple alignments of sequence data are straightforward if sequences are non-repetitive. However, if repeat length polymorphisms, which are common in parasite antigens, or insertions and deletion (indels) are found, then manual realignment needs to be done to ensure that gaps are correctly aligned, which can lead to an overestimate of the number of single nucleotide polymorphisms (SNPs). While repeat regions can be included in population genetic analyses, the expansion and contraction of repeat arrays does not impact on antigenic diversity as dramatically as amino acid changes (22, 131). However, by defining different alleles based on the number of repeats or in the case of indels, the presence or absence of a particular string of nucleotides, it is possible to predict whether such polymorphisms are modulated by immune selection.

Balancing selection, which is a result of immune selection pressure, favors the maintenance of diversity, with alleles at low to medium frequencies within populations, and balanced frequencies between populations (137). Polymorphic sites or regions that show such patterns are predicted to be under the influence of immune selection, and thus contribute to antigenic diversity. For some antigens under strong immune selection, similar alleles or clusters of alleles have been maintained across broad geographic areas (2, 110, 127, 129, 136, 146). The maintenance of moderate frequencies of allele clusters (and even individual alleles) across large geographic distances indicates that they represent distinct serotypes (2, 146).

In contrast, singleton polymorphisms and polymorphic sites with very low minor allele frequencies (MAFs) are indicative of deleterious mutations under purifying selection, or recent polymorphisms yet to increase in frequency. These polymorphisms will only be represented in a very small proportion of the parasite population. As the aim of diversity-covering vaccines is to encompass as many of the alleles (haplotypes) found as possible, several groups have chosen to exclude rare polymorphisms from population genetic analyses of vaccine antigens (110, 129, 145). Another cautionary note is that a large number of singleton polymorphisms in population datasets from the public databases may also indicate PCR artifacts, especially if the methodology includes a cloning step. If the validity of such data cannot be confirmed then it should be discarded from the comparative analyses.

### Analysis of the extent and distribution of diversity

In order to determine the diversity of the candidate antigen, both overall and within parasite populations from different geographic areas (e.g., village, district or country), genetic diversity should be estimated for all sequences combined and for each population. Genetic differentiation (e.g., Wrights fixation index, *F*_ST_) can be measured to determine whether allele frequencies vary and if not, populations from the same region or country can be considered as one population (2). Important diversity parameters to define for each population include the number and type of polymorphisms (SNP, indel, repeat length variation), the number and relative proportions of neutral (synonymous) and amino acid (non-synonymous) polymorphisms, and the number of haplotypes resulting from different combinations of amino acid polymorphisms. All of these statistics can be calculated using freely available population genetic analysis software and an excellent overview of these programs has been published (147). Conversion of polymorphisms to amino acid residues before haplotype definition ensures that complex codons and rare nucleotide polymorphisms within codons containing other more common polymorphisms are included. In any case, it is more appropriate to describe the amino acid haplotypes to predict serotypes, rather than the nucleotide haplotypes. Other more complex statistics such as the nucleotide and haplotype diversity can also be measured as an indication of the number and frequency of polymorphisms and haplotypes in the population, respectively; however, these are of more interest to the population geneticist than the vaccine developer.

To determine whether polymorphic sites are under the influence of balancing (immune) selection, statistical tests such as Tajima’s *D* can be performed using a sliding window approach, to identify specific domains or clusters of polymorphisms targeted by host immune responses (148). In addition, Wright’s *F*_ST_, has been applied to investigate whether allele frequencies at specific polymorphic sites, or gene domains, are balanced between populations (137). Polymorphisms under balancing selection and associated with immune escape will be non-synonymous, located in regions with a positive value of Tajima’s *D* and low inter-population *F*_ST_ values. However, sampling is extremely important as both the Tajima’s *D* and *F*_ST_ tests are based on allele frequencies. Populations must be of sufficient size so that the sampling error of the estimate is low, and panmictic (randomly mating) from the same geographic region, because any underlying geographic population structure may influence antigen allele frequencies (129, 136, 139, 140). Polymorphisms under balancing selection are also likely to have moderate MAFs, because the maintenance of diversity in the parasite population is advantageous for survival (137). The physical location of all polymorphisms identified as immune targets should then be mapped to the protein structure where possible, to help determine the potential functional relevance and implications of mutation at the site(s) identified, as has been successfully done for AMA1 [e.g., Ref. (126, 127)].

Determining the extent and distribution of immunologically relevant diversity for potential malaria vaccine candidates will help to determine the feasibility of whether a regionally or globally effective vaccine can be developed for the particular target. It is therefore important to focus the analysis to only those polymorphisms with the greatest likelihood of creating antigenic diversity to allow immune escape. Once polymorphisms predicted to be under balancing selection have been identified (see above and Figure 2), the distribution of unique haplotypes representing the predicted serotypes can be analyzed by determining haplotype frequencies in different geographic areas.

Different haplotypes will be related to differing degrees. Using network and clustering analyses, relationships between haplotypes from different populations, clusters of closely related haplotypes, and the extent and distribution of predicted serotypes can be investigated. By identifying the relationships among all of the different haplotypes in this way, the most distantly related alleles can be selected for further analysis or for inclusion in a vaccine to cover diversity. Previous studies have demonstrated the utility of these approaches to identify distinct clusters of alleles as a starting point to predict serotypes (11, 146, 149). These analyses identify the most distinct and common haplotypes and provide a basis upon which to select haplotypes that cover a large proportion of the population-wide diversity for a specific candidate antigen. The outcome of such analyses can help to determine: (i) whether it may be possible to cover all known diversity of the target sequence, (ii) the number of different haplotypes that would need to be included in a vaccine in order to cover diversity and (iii) the predicted efficacy of vaccine candidates. Inclusion of vaccine alleles in the analysis provides a reference point to estimate vaccine allele or serotype frequencies. The results of these analyses provide a simple diversity framework upon which to determine the parameters of allele specific and cross-reactive responses in pre-clinical studies (65, 127, 146) and to measure strain-specific efficacy in clinical trials (131). It is important to note that unlike other approaches that have clearly identified antigenic escape polymorphisms (126, 131), population genetic analyses provide a prediction, are simpler and less expensive, and can reduce the number of polymorphisms (and thus haplotypes) that need to be assessed to confirm their contribution to the serotype.

## Defining Antigenic Diversity by Monitoring the Dynamics of Diversity in Natural Parasite Populations

**Figure 3:**
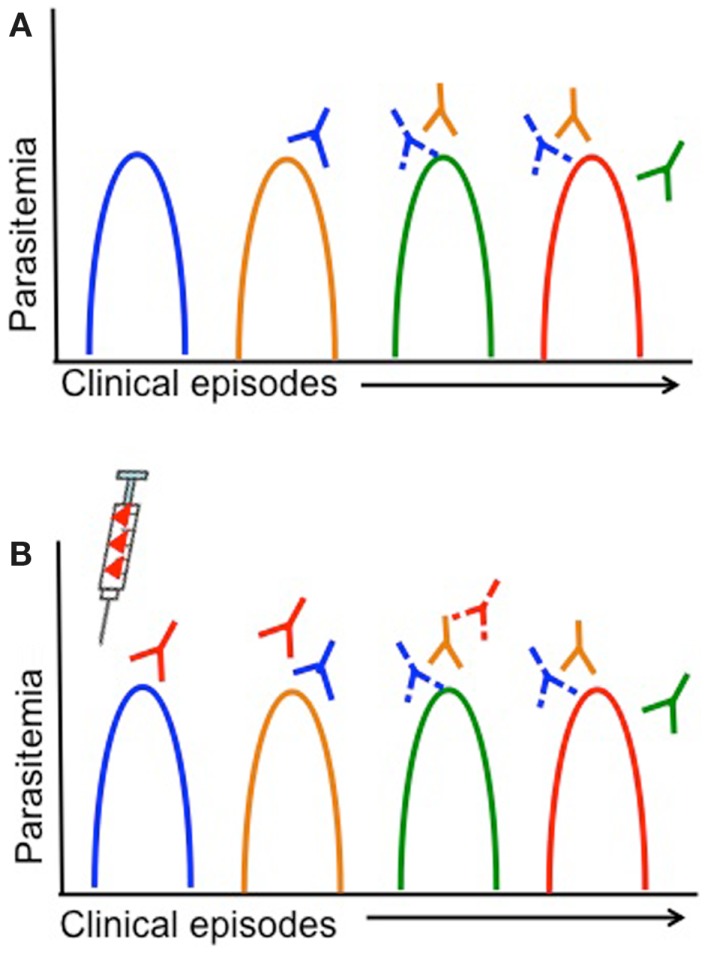
**Antigenic diversity, clinical malaria, and vaccine efficacy are shown**. Simplified overview of the impact of parasite antigenic diversity on the dynamics of natural infection and the efficacy of vaccines. Peaks in parasitemia correspond with different clinical episodes and colors indicate different serotypes. Strain-specific acquired antibodies are shown in corresponding colors some time after each clinical episode. Solid lines represent a strong antibody response, while dashed lines represent limited antibody responses. **(A)** Dynamics of natural infection with recurrent episodes of clinical malaria in an individual that acquires only strain-specific antibodies after being infected. As individuals experience different strains through natural infection (or vaccination), they acquire strain-specific antibodies and have a lower risk of having a clinical episode due to the same strain. **(B)** Lack of vaccine efficacy in a vaccinated individual due to antigenic diversity. The syringe indicates vaccination with a single serotype (red). If a single-strain vaccine is given at baseline, individuals are more likely to experience clinical episodes due to other strains (blue, yellow, green) than the vaccine strain, until antibody responses decrease.

Children living in malaria endemic areas are infected many times and have several episodes of clinical malaria before building up a large repertoire of antibodies that recognize a large number of strains (14, 15). Meanwhile, the rate of turnover of infections increases with age and is thought to be associated with the acquisition of antibodies to an increasing breadth of strains (150). Therefore, strains that children are yet to be exposed to pose a greater risk of clinical illness. Defining the dynamics of clinical infection in the context of parasite antigenic diversity may therefore provide insights into the specific genetic determinants required for antigenic escape, since the risk of a clinical episode due to infection with parasites representing a specific serotype would decrease after being exposed to that serotype (Figure 3A) (127, 138). Furthermore, analyses of strain-specific antibodies in conjunction with such analyses would confirm the status of immunity at a particular time point and allow associations to be made between gaps in the antibody repertoire and risk of a clinical episode (62, 98, 99, 151). Longitudinal studies in endemic regions that monitor children at regular time intervals for the presence of specific antibodies or the turnover of alleles at antigen loci could therefore be harnessed to identify the genetic determinants of antigenic diversity.

## Measuring Strain-Specific Efficacy in Vaccine Trials

Vaccines based on a single allele of the candidate antigen elicit strain-specific antibodies, so that vaccinated individuals continue to be at risk of infection with different strains but risk of infection with the vaccine strain is lower (Figure 3B) (22, 66, 131). Vaccine trials with strain-specific endpoints (i.e., infection with a particular parasite genotype) therefore represent a major opportunity to characterize the genetic determinants responsible for immune escape; however, this has been rarely attempted. Given the high diversity of most malaria vaccine candidates and the limited efficacy of single-strain vaccine candidates, for any candidate that elicits strain-specific responses it will be essential to measure strain-specific endpoints to ensure that antigenic escape is explored in vaccine testing. The advent of high throughput genotyping approaches (142, 152) has reduced the effort and funding required for molecular epidemiological studies, and therefore as long as relevant expertise is available, these investigations should be relatively simple to incorporate into a vaccine trial.

The primary efficacy endpoint in clinical trials of malaria vaccines has included a range of measurements such as time to first infection, occurrence of a clinical episode (mild and severe), and parasite density. However, only a few malaria vaccine trials have determined whether efficacy against vaccine or closely related strains has been achieved (22, 92, 131). Below, we cover some molecular epidemiological analyses that could be considered in the different stages of a vaccine trial.

### Vaccine trial reconnaissance

The low prevalence of vaccine alleles within natural populations has been proposed to limit vaccine efficacy (2, 11, 129, 138). Furthermore, a low prevalence of the vaccine allele will make it extremely difficult to measure allele specific efficacy, since changes may be very small and therefore require exceedingly large sample sizes (92). Therefore, even before a vaccine trial begins in a particular geographic area, molecular epidemiological surveys will be important to assess baseline population genetic characteristics, and in particular the allele- (or if known, the serotype-) frequencies to ensure that the vaccine to be tested is representative of the target parasite population.

### Strain-specific endpoints

In addition to measuring strain-specific endpoints by determining the time to first clinical episode with vaccine and non-vaccine alleles, the analysis of outcomes in control and vaccine groups provide an opportunity for more detailed analyses that can provide key insights into the complexities of parasite antigenic diversity (92, 127, 153). In principle, the same analyses can be done in longitudinal studies to achieve the same goal. These analyses will provide further functional evidence of antigenic escape polymorphisms, thus allowing refinement of the serotype. Different types of analyses that have been used to measure strain-specific efficacy in vaccine trials include:

(i) *measuring strain-specific protection* by assessing risk of clinical episode associated with parasites carrying the vaccine-allele compared to those carrying any non-vaccine allele;
(ii) *measuring cross-strain protection* by assessing risk of clinical infection with parasites carrying the vaccine-allele compared to those carrying different non-vaccine alleles;
(iii) *measuring vaccine-mediated selection* by:
  - comparison of allele or haplotype frequencies before and after vaccination in the different vaccine groups;
  - comparison of the incidence of infection with parasites carrying different haplotypes and individual polymorphisms before and after vaccination;
(iv) *assessment of vaccine-mediated strain-specific natural immunity* by measuring antibodies to vaccine alleles in vaccine and control groups.

### Vaccine-mediated selection and antigenic escape mutants

The development and licensing of an effective malaria vaccine will require sustained and intensive surveillance to monitor for vaccine-mediated selection of non-vaccine alleles and to ensure that vaccine alleles are common enough for vaccines to remain effective. Regular population genetic surveys will be required in order to monitor allele frequencies, to ensure that alleles contained within the vaccine continue to represent the diversity of the parasite population and that new, potential antigenic escape mutants have not emerged. A genetic surveillance system would be difficult and costly to implement, but the development of low cost rapid assays to quickly genotype parasite isolates would facilitate such an approach (152). The need for simple and informative surveillance tools also highlights the importance of gaining knowledge into the precise determinants of antigenic diversity in any antigen to be included in a licensed malaria vaccine.

## Conclusion

The trials and tribulations of malaria vaccine development have reached a critical juncture. The first licensed malaria vaccine is almost at hand and children in African countries stand to benefit greatly from its availability. However, this vaccine is only partially effective against the symptoms of malaria, not infection, and provides only short-lived protection (23). As a result of the success of the reinvigorated global malaria eradication program, many previously highly endemic countries no longer have high burdens of disease. By 2030, the goal is to have a second-generation vaccine(s) that can provide broad and long-lived protection against multiple species and diverse strains. Parasite antigenic diversity, one of the major reasons for the failures of past candidate malaria vaccines, remains a barrier to the efficacy of subunit and potentially even whole parasite vaccine approaches (48), but there is light at the end of the tunnel: this review demonstrates how it may be possible to overcome parasite antigenic diversity. Successful identification of the critical genetic determinants of the serotype for one leading vaccine candidate, AMA1 (126, 127, 131) and proof that multivalent vaccines representing the majority of the diversity of this antigen can generate broad antibody responses further support the development of effective multi-allele vaccines and suggest that antigenic diversity in malaria may be overcome (65, 132). By consolidating the vast knowledge gained on the genetic diversity of candidate antigens, by harnessing high throughput genotyping tools and with careful design of longitudinal studies and vaccine trials, it is highly likely that it will also be possible to identify the critical genetic determinants underlying antigenic diversity (i.e., the serotype) for any candidate malaria vaccine antigen.

## Author Contributions

Alyssa E. Barry and Alicia Arnott wrote the paper, collated data, and prepared figures.

## Conflict of Interest Statement

The authors declare that the research was conducted in the absence of any commercial or financial relationships that could be construed as a potential conflict of interest.
